# Outcomes of surgical treatment for upper urinary tract transitional cell carcinoma: Comparison of retroperitoneoscopic and open nephroureterectomy

**DOI:** 10.1186/1477-7819-6-3

**Published:** 2008-01-15

**Authors:** Tawatchai Taweemonkongsap, Chaiyong Nualyong, Teerapon Amornvesukit, Sunai Leewansangtong, Sittiporn Srinualnad, Bansithi Chaiyaprasithi, Phichaya Sujijantararat, Anupan Tantiwong, Suchai Soontrapa

**Affiliations:** 1 Division of Urology, Department of Surgery, Faculty of Medicine Siriraj Hospital, Mahidol University, Bangkok, Thailand

## Abstract

**Objectives:**

To determine the surgical and oncologic outcomes in patients who underwent retroperitoneoscopic nephroureterectomy (RNU) in comparison to standard open nephroureterectomy (ONU) for upper urinary tract transitional cell carcinoma (TCC).

**Patients and methods:**

From April 2001 to January 2007, 60 total nephroureterectomy were performed for upper tract TCC at Siriraj Hospital. Of the 60 patients, thirty-one were treated with RNU and open bladder cuff excision, and twenty-nine with ONU. Our data were reviewed and analyzed retrospectively. The recorded data included sex, age, history of bladder cancer, type of surgery, tumor characteristics, postoperative course, disease recurrence and progression.

**Results:**

The mean operative time was longer in the RNU group than in the ONU group (258.8 versus 190.6 min; p = 0. < 001). On the other hand, the mean blood loss and the dose of parenteral analgesia (morphine sulphate) were lower in the RNU group (289.3 versus 313.7 ml and 2.05 versus 6.72 mg; p = 0.868 and p = 0.018, respectively). There were two complications in each group. No significant difference in p stage and grade in both-groups (p = 0.951, p = 0.077). One patient with RNU had lymph node involvement, three in ONU. Mean follow up was 26.4 months (range 3–72) for RNU and 27.9 months (range 3–63) for ONU. No port metastasis occurred during follow up in RNU group. Tumor recurrence developed in 11 patients (bladder recurrence in 9 patients, local recurrence in 2 patients) in the RNU group and 14 patients (bladder recurrence in 13 patients, local recurrence in 1 patient) in the ONU group. No significant difference was detected in the tumor recurrence rate between the two procedures (p = 0.2716). Distant metastases developed in 3 patients (9.7%) after RNU and 2 patients (6.9%) after ONU. The 2 year disease specific survival rate after RNU and ONU was 86.3% and 92.5%, respectively (p = 0.8227).

**Conclusion:**

Retroperitoneoscopic nephroureterectomy is less invasive than open surgery and is an oncological feasible operation. Thus, the results of our study supported the continued development of laparoscopic technique in the management of upper tract TCC.

## Background

The standard surgical procedure to treat upper urinary tract transitional cell carcinoma (TCC) is open nephroureterectomy (ONU) with bladder cuff excision. However, the morbidity of open surgery (e.g. severe pain and prolonged convalescence) is inevitable. In 1991, Clayman firstly described the technique of laparoscopic nephroureterectomy (LNU), which was soon replicated by various authors worldwide [1]. Recently, LNU through the transperitoneal or retroperitoneal approach has been used to treat upper urinary tract TCC, with reduced morbidity [2]. Although the many other benefits of LNU are clear, the application of these techniques to the treatment of cancer raises issues relating to oncologic safety. Up to date, most studies have shown the oncologic outcomes of LNU comparable to ONU groups [3,4]. However, few reports with adequate follow up in upper tract TCC patients after retroperitoneoscopic nephroureterectomy (RNU) have been published [5-7]. To determine whether the surgical and oncologic outcomes of RNU is at least equivalent to that of ONU, we present our 7 years experience of RNU with open bladder cuff excision, compared with patients after ONU, in upper urinary tract TCC treatment.

## Patients and methods

From April 2001 to January 2007, 60 patients underwent total nephroureterectomy with bladder cuff excision for upper tract TCC at Faculty of Medicine Siriraj Hospital. According to the decision of the surgeon's preference, 31 patients were treated with RNU, and 29 patients with ONU. In all patients, the surgery was performed completely extraperitoneal with open bladder cuff technique. Upper tract TCC was diagnosed by intravenous urography, retrograde pyelography, computed tomography of the abdomen, magnetic resonance imaging, and ureteroscopy with or without biopsy. Preoperative cystoscopy and radiologic examinations were performed to rule out metastasis and concomitant bladder cancer.

LNU was performed using the retroperitoneal approach. The patient was placed in a lateral position. After a retroperitoneal working space had been created, the pneumoretroperitoneum was maintained with carbon dioxide gas at 10 mmHg. Three or four trocars were inserted in the usual manner. The posterior peritoneum was mobilized medially so that dissection of Gerota's fascia and the renal pedicle could be fully performed. After the lymphatic channels around the renal pedicle were excised to expose the renal artery, this artery was isolated, clipped and divided. The renal vein was mobilized and secured with clips. Caudally, the fatty tissue around the ureter was divided at the level of the iliac vessels crossing. Finally the kidney was completely mobilized. Lymphadenectomy was performed at surgeon's discretion. The patient position was then changed to supine. An approximately 7 cm long Gibson's incision was made, and the distal ureter with a bladder cuff specimen was removed en bloc without opening the urinary tract. If the cancer was located in the mid or distal ureter, lymphadenectomy was consecutively performed around the lesion.

The standard ONU was performed using a flank incision. The distal ureter management was performed as standard technique. All patients with concomitant bladder tumor were underwent transurethral resection concomitantly.

All patients with proven nodal disease were counseled for adjuvant therapy. Patients have a follow-up cystoscopy every 3 months in the first 2 years, every 6 months in the following 3 years and annually after 5 years.

We retrospectively reviewed our database and extracted data on the following variables: sex, age at diagnosis, history of previous bladder cancer, type of surgery, complications, tumor characteristics, postoperative course, disease recurrence and disease progression.

The comparison between the two groups was carried out using the Mann-Whitney U test and Fisher's exact test. Time to recurrence was evaluated from the date of surgery. Recurrence free survival was defined as the interval from surgery to the first tumor recurrence, the detection of distant metastases or the end of the study. Survivals were analyzed by the Kaplan-Meier method. To assess the effect of type of surgery on time to recurrence after adjusting for the effects of pathological stage and grading, a Cox's proportional hazard model was fitted. For all statistical tests, P < 0.05 was considered to indicate a significant difference.

## Results

The characteristics of the patients who underwent RNU and ONU are shown in Table 1. There was no significant difference in mean age (p = 0.353), operative side (p = 0.796), tumor location (p = 0.233), and concomitant or history of bladder cancer (p = 0.599).

**Table 1:** Patient characteristics

**Variable**	**Number (%) or Mean (Min-Max)**		
	
	**RNU (N = 31)**	**ONU (N = 29)**	**P Value**
Age, years	63.8 (26–79)	66.8 (39–88)	0.353
Sex			
Male	11 (35.5)	22 (75.9)	
Female	20 (64.5)	7 (24.1)	
Side			
Left	18 (58.1)	15 (51.7)	0.796
Right	13 (41.9)	14 (48.3)	
Tumor location			
Renal pelvis	14 (45.2)	10 (34.5)	0.233
Ureter	13 (41.9)	10 (34.5)	
Multifocal	4 (12.9)	9 (31)	
Concomitant or history of bladder cancer	11 (35.5)	13 (44.8)	0.599

A comparison of the perioperative parameters between the two groups is shown in Table 2. No significant differences were founded in blood transfusion, mean time to first diet, length of indwelling urethral catheter, and hospital stay. The mean operative time was significant longer in the RNU group (p = <0.001). However, although not to a significant extent, the mean blood loss tended to be less in the RNU group (289 vs. 313 ml).

**Table 2:** Surgical results

**Variable**	**Mean (Min-Max) or Number (%)**		
	
	**RNU (N = 31)**	**ONU (N = 29)**	**P Value**
Operative time (min)	258.87 (90–425)	190.69 (105–360)	<0.001*
Blood loss (ml)	289.35 (100–800)	313.79 (50–800)	0.868
Blood transfusion	6 (19.3)	7 (24.1)	0.758
Time to first diet (days)	1.13 (1–2)	1.10 (1–2)	1.000
Time to remove of urethral catheter (days)	6.81 (2–16)	6.24 (1–11)	0.727
Hospital stay (days)	9.32 (6–20)	8.69 (5–13)	0.890
Parenteral analgesia			
Morphine sulphate (mg)	2.05 (0–10)	6.72 (0–35)	0.018*
Complication			
Ischemic heart disease	1	0	
Urinary tract infection	1	0	
Re-explor (bleeding)	0	1	
Urinoma	0	1	

Additionally, the dose of parenteral analgesia was significantly reduced in RNU group (p = 0.018). Complications developed in 2 patients of each group. In the RNU group, one patient had ischemic heart disease which required coronary angiography. Another patient had postoperative urinary tract infection and required parenteral antibiotic with prolonged hospital stay. In the ONU group, one patient had postoperative bleeding which required open surgery to stop bleeding. Another patient had an urinoma at perivesical space and required surgical drainage.

The oncologic results are shown in Table 3. There were no statistical difference in tumor stage and grade in both groups (p = 0.951 and p = 0.077, respectively). Lymphadenectomy was performed in 20 patients (64.5%) with RNU and 9 patients (31.0%) with ONU groups. One patient in each group was found to have a single lymph node micrometastasis. Both patients were managed conservatively due to refuse chemotherapy and further follow-up to 30, 31 months respectively showed no evidence of disease recurrence. Another two patients in ONU group had multiple lymph node metastasis. One patient developed bone metastasis after 8 months despite adjuvant systemic chemotherapy. Another patient, with large persisting lymph node at resection site, died of to tumor progression after 7 months. It was noted that this patient had no adjuvant therapy due to poor performance status.

**Table 3:** Oncologic results

**Variable**	**Number (%) or Mean (Min-Max)**		
	
	**RNU (N = 31)**	**ONU (N = 29)**	**P Value**
Pathologic stages			
T1	16(51.6)	13 (44.8)	0.951
T2	10 (32.3)	12 (41.4)	
T3	4 (12.9)	4 (13.8)	
T4	1 (3.2)	0 (0.0)	
Grade			
Low	18 (58.1)	10 (34.5)	0.077
High	13 (41.9)	19 (65.5)	
Node	20 (64.5)	9 (31.0)	
Negative	19 (95)	6 (66.7)	0.076
Positive	1 (5)	3 (33.3)	
Recurrence	11 (35.4)	14 (48.2)	0.300
Bladder	9 (29.0)	13 (44.8)	0.285
Local	2 (6.4)	1 (3.4)	
Metastasis	3 (9.7)	2 (6.9)	1.000
Follow up time (months)	26.4 (3–72)	27.9 (3–63)	0.534
2 yr. disease specific survival	86.3%	92.5%	0.8227
2 yr. overall survival	86.3%	83.3%	0.8628

**Figure 1:**
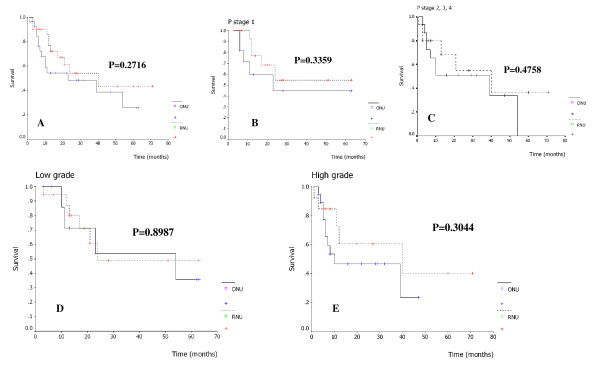
Recurrence free survival according to surgical procedure (A), stage (B, C), grade (D, E).

**Figure 2:**
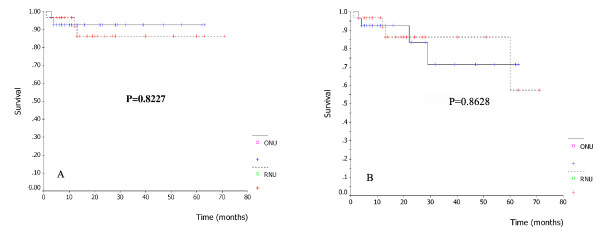
Disease specific survival (A), overall survival (B) according to surgical procedure.

There was no port site metastasis occurred during follow up in RNU group. Bladder cancer recurrence occurred in 9 patients (29%) in the RNU group and 13 patients (44.8%) in the ONU group. No statistically significant difference was observed (P = 0.285). Local recurrence developed in 2 patients in the RNU group and 1 patient in the ONU group, in 2 of whom distant metastases in the lung and bone were detected simultaneously. All three patients had a negative surgical margin on histopathological examination. The metastasis rate was 9.7% (3/31) after RNU and 6.9% (2/29) after ONU (p = 1.00). The median time to metastasis was 12 months (range 6–14) and 14 months (range 8–20) in the RNU and ONU groups, respectively. For the RNU group, three patients died of distant metastasis (two in the liver, one in the lung) and one patient died of cardiac disease during the follow up period. For the ONU group, two patients died of disease progression (one in the lung, one in the lymph node) and two patients died from other causes unrelated to tumor. The median time to recurrence was 40 months (range 3–71) and 23 months (range 3–63) in the RNU and ONU groups, respectively. The prognostic factors studied by multivariate analysis given in Table 4. Analysis results revealed that even though ONU seemed to have a higher risk of recurrence than RNU (HR = 1.50, 95% CI = 0.67, 3.35) there was no statistical difference (p = 0.323). There was also no significant effect of stage (stage 2: HR = 1.15, p = 0.776; stage 3: HR = 2.58, p = 0.144) and grade (High: HR = 1.21, p = 0.701) on recurrence. For recurrence free survival analysis, we found no statistically significant difference between the two procedures (p = 0.2716) (Fig. 1A). Additionally, we found no statistically significant difference in recurrence free survival curves between the two procedures in terms of p stage and grade (Fig. 1B–E). The mean follow up time of the RNU group and the ONU group was 26.4 months (range 3–72) and 27.9 months (range 3–63) respectively. No significant difference was found between the two procedures with regard to disease specific and overall survival (Fig. 2A, B). The 2 years disease specific survival rate was 86.3% in the RNU group and 92.5% in the open group (P = 0.8227). The corresponding 2 years overall survival rate was 86.3% and 83.3% (P = 0.8628).

**Table 4:** Results of Cox's regression

	**b**	**Hazard ratio (HR)**	**95% CI of HR**	**p-value**
ONU	0.406	1.50	0.67, 3.35	0.323
Stage 2	0.139	1.15	0.44, 3.00	0.776
Stage 3	0.947	2.58	0.72, 9.18	0.144
High grade	0.189	1.21	0.46, 3.17	0.701

## Discussion

Laparoscopic nephroureterectomy was developed in an effort to reduce the morbidity of the surgical management. Indeed, several investigators have recently suggested their benefit for patient recovery with disease control comparable to that of traditional open surgery [2-4]. The mean oral diet day, urethral catheter time, and hospital stay were equivalent in the both groups in our series. However, the operative time was longer in the laparoscopic groups. On the other hand, the blood loss and the dosage of analgesia were lower after laparoscopic nephroureterectomy. In a literature review of 1365 nephroureterectomy patients, Rassweiler et al. reported the operative time (277 vs. 220 min) and the blood loss (241 vs. 463 ml.) comparing between the laparoscopic series and open series [2]. These findings correspond to our results and support the effectiveness of laparoscopic procedure compared with the standard open procedure.

Laparoscopic nephroureterectomy can be performed via a transperitoneal or retroperitoneal access. We used the retroperitoneal approach. Although the operating space is smaller and a more skilled technique is required than with the transperitoneal approach, the advantage of retroperitoneal approach in avoiding intraabdominal injury and tumor spillage into intraabdominal cavity are our consideration. Rouprêt *et al*. reported the complications of colonic injury after transperitoneal LNU [4]. We found no complication of intraabdominal injury and two minor complications after retroperitoneal LNU in our series. These finding confirmed the benefit of retroperitoneal approach and a feasible technique for LNU. Additionally, the technique of ureterectomy and bladder cuff excision has not been standardized yet. A number of minimal invasive approaches to the distal ureter such as endoscopic stripping or pluck-off techniques have been reported [8-11]. However, these endoscopic techniques have a greater risk of local recurrence and stone formation in the staple lines [12]. We prefer open distal ureterectomy and bladder cuff excision. This method avoids the risk of urinary leakage and allows for intact specimen removal. We believed this will not adversely affect patient's recovery compared with the endoscopic approach. Furthermore, there are no contraindications such as ureteral tumors or periureteral fibrosis due to previous surgery, irradiation or inflammatory pelvic disease [13]. The worldwide reported bladder recurrence rate was 9–48% with different methods for controlling the bladder cuff [2,14,15]. In our series, the bladder recurrence rate (29%) after RNU was within the reported range. In addition, the problem of port site metastasis in laparoscopic procedure is important. Rassweiler et al. reported that six port site metastasis in 377 (1.6%) analyzed patients following laparoscopy were recognized [2]. Recently, Schatteman *et al*. reported another three cases of port metastasis after laparoscopy [16]. In most cases, extraction of the specimen was performed without an organ or with a torn organ bag. In our series, no case of port site metastasis was observed during the follow up period. We routinely avoid the use of harmonic scalpel for tissue dissection which might be an origin of tumor cell spreading as previously described [17] and we retrieved the intact specimen via the open wound.

The indication for laparoscopic nephroureterectomy in upper tract TCC is not yet well defined. Although most authors still recommended that high stage and grade tumors should be contraindications to LNU [2,3,5]. Recently in 2007, Muntener *et al*. reported oncologic outcome after LNU with a median follow up time of 74 months and supported the LNU as the standard of care for high grade or high stage upper tract TCC [18]. In our series, we found no statistically significant difference in recurrence free survival curve between both procedures in terms of tumor grade and stage (Fig. 1B–E). However, we believe that the indication tend to increase as surgical skill developed in laparoscopic treatment and we could have identified additional candidates with high grade or high stage tumor for LNU if accurate staging with preoperative imaging and biopsy had been done.

McNeill et al. reported favorable long term outcomes after LNU compared with ONU; however, information on nodal status was available in only 4% of cases [19]. Klinger et al. found micrometastasis in 14.3% (2 of 14) of clinical No patients and advised to perform lymphadenectomy routinely for staging purpose [17]. In our series, lymphadenectomy was performed in 48.3% (29/60) of cases. We had no definitive criteria for choosing the surgical procedure, including the indication for lymphadenectomy, which might affect the results of treatment. We found micrometastasis in 2 patients and these patients are still alive until the last follow up time. However, the prospective randomized study is needed to support the benefit and efficacy of routine laparoscopic regional lymphadenectomy.

In 2000 Gill et al. reported retroperitoneoscopic nephroureterectomy with bladder cuff excision through a transvesical approach and at a mean follow up of 11 months the cancer specific survival rate was 97% in the LNU group [20]. Hsueh *et al*. reported Hand assisted RNU with open bladder cuff excision compare to ONU [7]. The study showed no significant difference in terms of the disease specific and overall survival rate between the two groups. In 2007, Manabe *et al*. reported oncologic outcome of LNU with the same surgical approach as in our study. The study showed the 2 years disease specific survival rate were similar in both groups (85.2 vs 87%) [21]. The worldwide reported disease survival was 72–95% with different methods for LNU and distal ureter management [16,17,22]. In the present series shows a 2 years disease specific survival of 86.3% which is comparable to literature data. No significant difference in disease specific and overall survival curve were found between both procedures. These results confirmed the oncologic safety of retroperitoneoscopic nephrectomy compared with the standard ONU.

## Conclusion

The retroperitoneoscopic nephroureterectomy with open bladder cuff excision seems to be a safe alternative treatment for upper urinary tract TCC and offers the advantages of laparoscopic procedure. From the oncologic stand point, it is not associated with an increased risk of tumor recurrence compared with the standard open neprhoureterectomy. Because of limitation in retrospective study, thus a true prospective and continued evaluation of longer follow up data are needed before RNU should become the new standard of care for the upper tract TCC.

## Competing interests

The author(s) declare that they have no competing interests.

## Authors' contributions

TT conceived and participated in the study performed statistical analysis interpreted the data and prepared the draft manuscript. TA and BC helped in interpretation of data and preparation of the manuscript; CN, SL, SIS participated in acquisition of data and preparation of manuscript; PS, AT and SUS helped designing the study and manuscript preparation. All authors read and approved final manuscript for publication.
